# Penetrating orbit injury: challenge to emergency medicine

**DOI:** 10.1186/1756-0500-6-493

**Published:** 2013-11-28

**Authors:** Gyanendra Malla, Rabin Bhandari, Pramendra Prasad Gupta, Rajani Giri

**Affiliations:** 1 Head of Department, Dharan, Nepal; 2 Department of General Practice and Emergency Medicine, B.P.Koirala Institute of Health Sciences Dharan-18, Dharan, Nepal

## Abstract

**Background:**

Penetrating orbital injuries pose a serious threat to vision, ocular motility, and in some cases, life. The setting and causes of eye injury are diverse, but previous studies have demonstrated that the risk and type of injury is often correlated with age, gender, and race. Pediatric ocular injury is often accidental and may be preventable. A focused history and prompt ocular examination are essential to immediate management.

**Case presentation:**

This article describes a case of protruding foreign body-related penetrating orbit injury with a retained foreign body in a 4-year-old male from a town in the eastern part of Nepal. The child presented to the emergency with foreign body in situ without receiving any pre emergency care without any medical attendance. The patient was managed with non-operative removal of foreign body in the emergency. The case discussion will review the initial presentation, examination, resultant management decisions, and final outcome.

**Conclusion:**

Foreign body presentations may be diverse and non-operative management may be considered in selected cases. Resource availability and conditions at presentations may also influence the management decisions. This case presentation has described such a scenario in developing country like Nepal and is expected to be interest across various medical specialties.

## Background

Ocular injury is a frequent, preventable cause of visual impairment, with a lifetime prevalence of 19.8% [1]. Pediatric patients account for a large proportion of these injuries (8-14%). Pediatric ocular injury is usually accidental and uniocular [2,3]. Assessment of these injuries in pediatric patients is challenging given the difficult examination.

This case report outlines the presentation of a child with a penetrating orbitocranial injury. Although similar cases are published, this 4-year-old child presented fully conscious, with an orbitocranial foreign body and massive intracranial penetration, after traveling far on rough roads with relatives supporting the large protruding object (approx. wt. 1 kg and length of 9 cm). This case demonstrates a positive outcome for a potentially catastrophic injury in a resource limited environment.

## Case report

**Figure 1:**
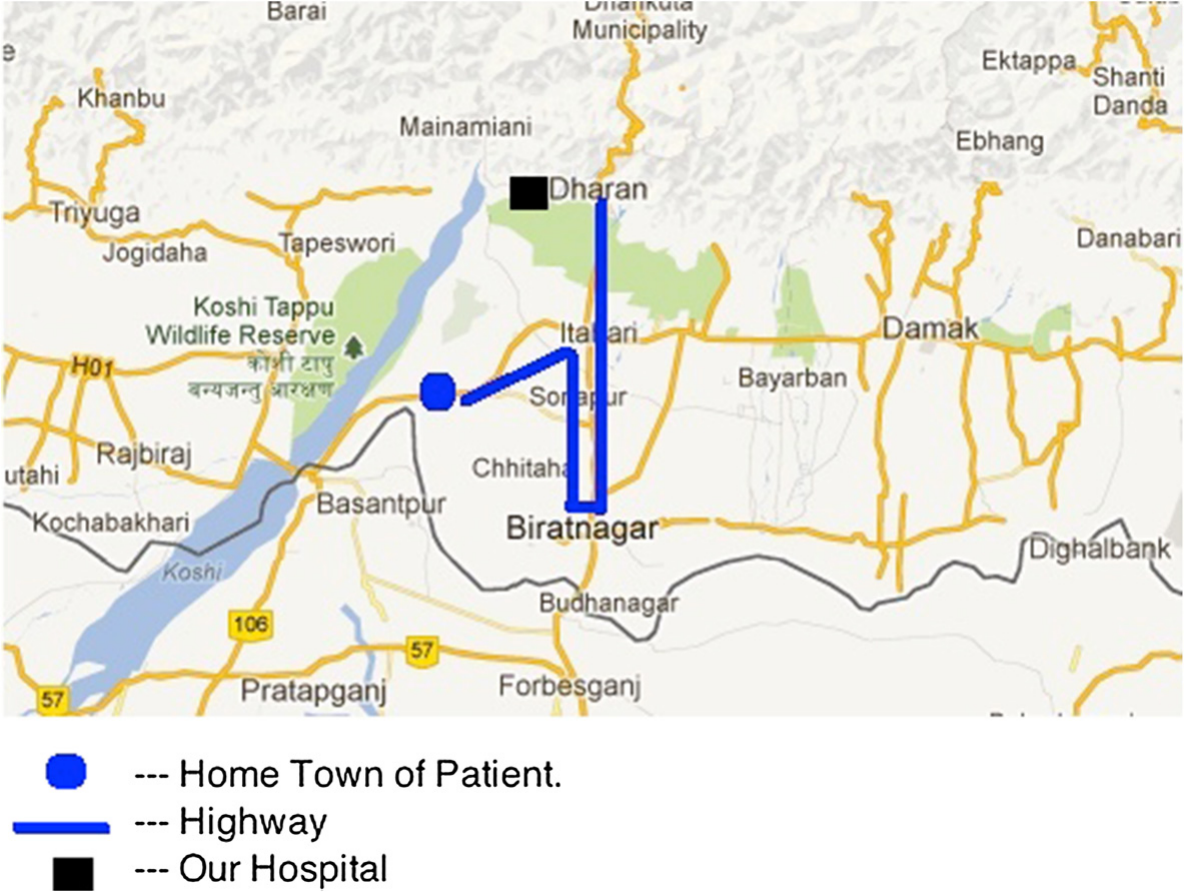
Home town patient, highway and our hospital.

A 4 year old male presented to the emergency department with the history of falling off a ladder 3 hours prior. He landed on his face on a wooden spike that embedded itself in the right orbit. His local hospital is not equipped to deal with ocular emergencies so he was transported by car for approximately 40 km to an eye hospital. Unfortunately, no anesthetist was available and the patient was referred to our emergency department. No treatment was instituted prior to referral. To reach our hospital, the patient had to backtrack 22 km and travel an additional 18 km, 40 km in total over rough roads (Figure 1).

**Figure 2:**
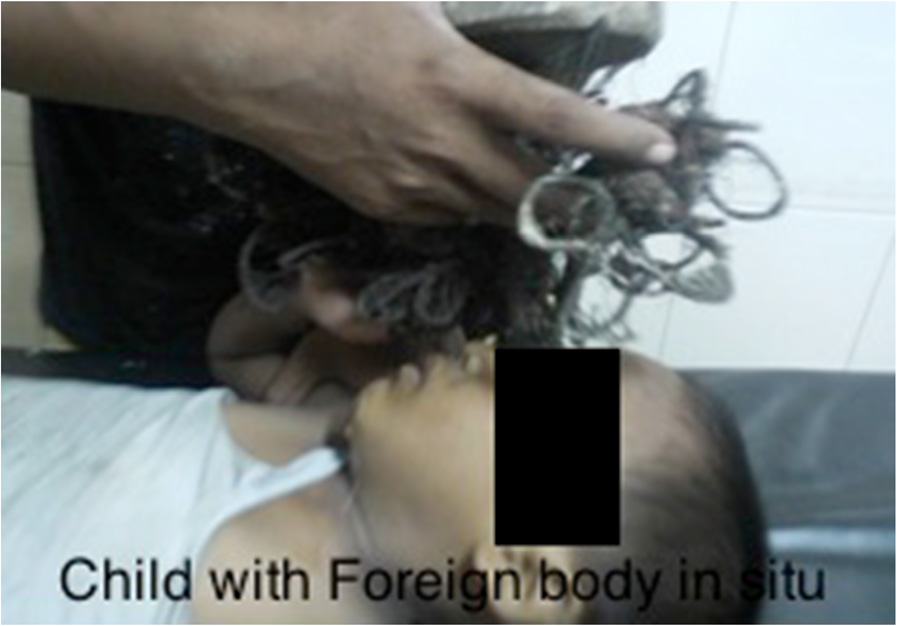
Child with foreign body in situ.

On arrival in our emergency department the child was fully conscious. He was hemodynamically stable with no airway or respiratory compromise. He had no past medical history and was not taking any regular medication. History revealed he was up to date with his immunizations, but history was considered unreliable. He lived with his parents and younger brother in a town in Eastern Nepal. On further examination, no other injuries were found specifically the cervical spine. Gross neurological examination was normal. The foreign body, still in situ, was a sharp wooden peg. It had penetrated the right orbit along the medial side. The cumbersome protruding object constituted a rock and a wire mesh attached to the wooden peg. The weight (approx. 1 kg) and shape of the object made stabilization of the object particularly difficult. It was impossible to evaluate the eye further due to the eye being obscured by the protruding object (Figure 2).

**Figure 3:**
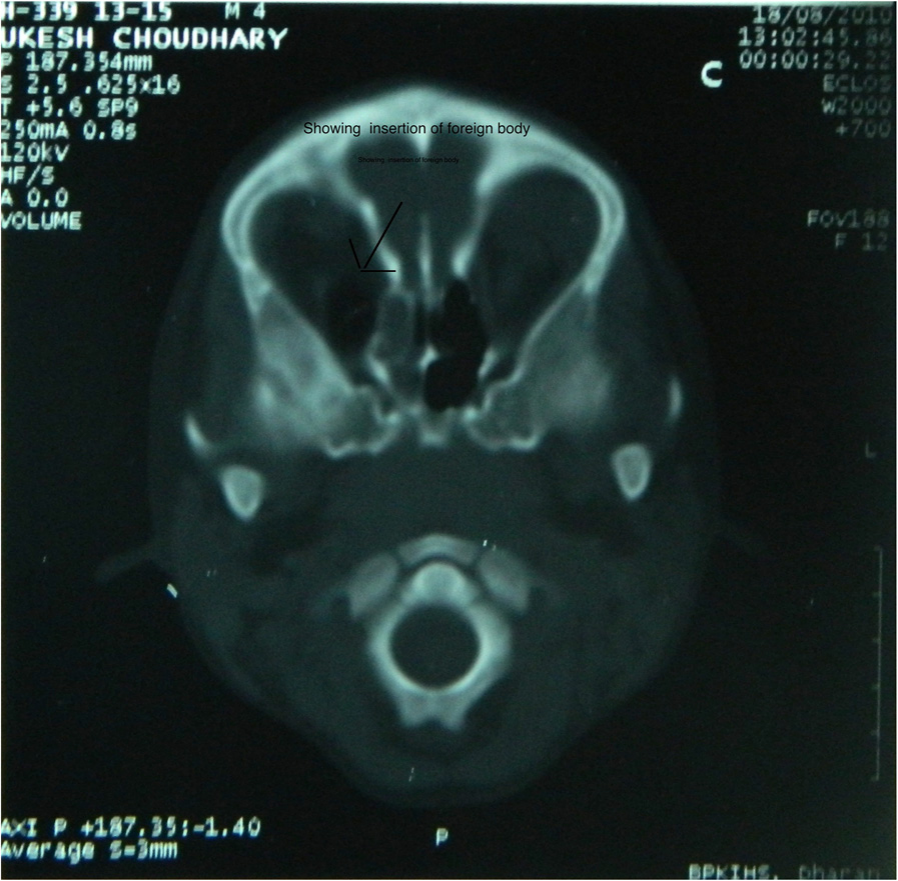
Showing insertion of foreign body.

**Figure 4:**
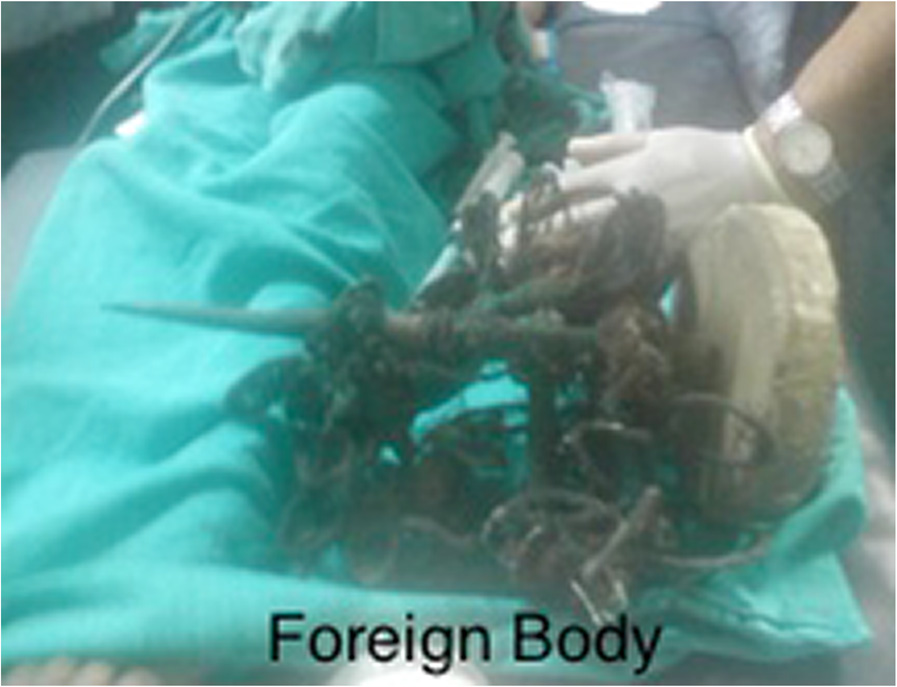
Foreign body.

Intravenous access was established. Pain was managed with morphine and prophylactic antibiotics were initiated. CT scan was arranged. Patient was sedated with ketamine and midazolam. The patient was transferred to CT scan with the relative supporting the foreign body all the time. The contrast CT was initially reported by a radiology resident as follows, “foreign body lateralizing globe and penetrating through the medial part of the orbit extending through orbital apex to middle cerebral fossa and just impinging right cerebellar hemisphere. Minor contusions in right cerebellum and hemi sinus ethmoidal sinus are suspected. The length of the penetrating part was around 9 cm” (Figure 3). Upon immediate ophthalmology consultation, a decision to remove the foreign body was made. Again, anesthetists were unavailable for timely removal of the foreign body as they were busy in theatre. Due to the time delays pre presentation, it was decided that removal of the foreign body should occur as quickly as possible. The ophthalmologist and the emergency physician removed the foreign body, with the aid of sedation (ketamine, morphine and thiopentone). A lateral canthotomy was performed (Figure 4).

**Figure 5:**
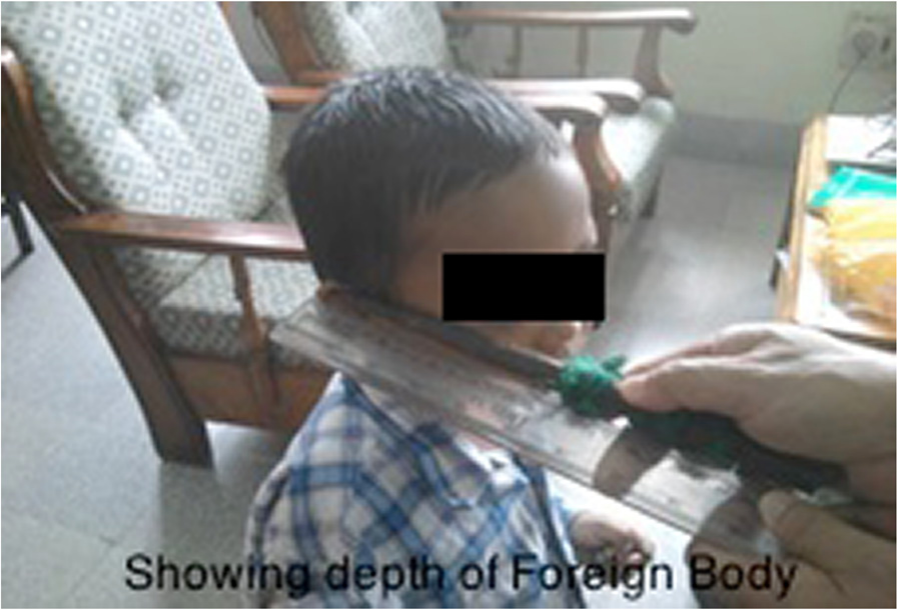
Showing depth of foreign body.

After removal of the foreign body, the eye examined as follows: axial proptosis, mid dilated pupil, clear lens, and clear media. There was no evidence of globe penetration. Pilocarpine drops were instilled and ciprofloxacin ointment used before padding and bandaging the eye. Oral acetazolamide was started. Otolaryngologist and Surgical specialties were consulted with no further management advised. There was no neurosurgeon at the hospital available for consultation. The patient remained in the emergency department for observation (Figure 5).

At 48 hours the patient remained neurologically intact. His dressing was removed and his eye examined as follows: subconjunctival hemorrhage, clear cornea, mid dilated pupil, clear lens. Patient had no light perception in the right eye. A repeat CT showed no deleterious changes. On discharge on day 3, patient was advised to follow up with a neurosurgeon in another city.

3 month follow up revealed the following examination: mild proptosis, no blinking, no eye movement, pupil dilated and non-reactive, cornea clear and no light perception. Fundoscopy revealed a pale optic disc. Eye taping was advised. At 6 months, he had regained some blinking, but had not recovered any light perception.

## Discussion

Evidence of penetrating orbitocranial injury escaping with minimal or no serious complications has been noted in the literature. The pattern of injury generally reflects the type of society one lives in. On presentation to the emergency department, the emergency physician should co-ordinate the multidisciplinary care of a patient with a penetrating eye injury [4]. The orbit is shaped like a cone; hence penetrating objects are directed towards the apex and usually pass through the superior orbital fissure and optic canal to enter the intracranial space. [5,6]. Complications range from traumatic cranial neuropathies to potentially fatal intracranial injuries [7]. Ocular complications include optic nerve damage with resultant visual loss, extra-ocular muscle paralysis secondary to direct muscle trauma or nerve damage, proptosis or macular edema [8]. Finally, given the orbit’s close proximity to the paranasal sinuses, infection, specifically abscess formation is a common complication. Even if the cranium has not been breached, the presence of orbital hematomas, abscesses, optic nerve sheath hematomas and some foreign bodies (organic material, copper) are deemed true emergencies [9,10]. Wooden foreign bodies require expeditious removal and broad spectrum antibiotic coverage to reduce infective complications. The decision to remove the foreign body in Emergency Department in this case was made taking this into consideration, together with the fact that a theatre and anesthetist were not available. Referral of this patient to another hospital exposed the patient to further transport risk with an unstable, cumbersome foreign body still in situ.

Ketamine is favored in developing countries due to its availability, safe profile, ease of use, analgesic, sedative and amnesic properties while maintaining airway muscle reflexes and tone. It is safe to use in children and requires minimal monitoring [11,12]. This justifies our use in low doses together with midazolam. The sedation was supervised by a consultant general practitioner who supervises the emergency department.

## Conclusion

Penetrating ocular foreign bodies can have varied presentations. Transfer for investigation of these patients can be extremely challenging, especially in children. Resource availability, time constraints and complications guide the management. This case shows that successful removal of a penetrating oculo-cranial foreign body by non-operative methods can be done in the emergency department in carefully selected patients.

## Consent

Written informed consent was obtained from the patient’s mother (as patient is a minor) for the publication of the case report and the accompanying images. A copy of the written consent is available for review by the Editor-in-Chief of this journal.

## Competing interests

The authors declare that they have no competing interests.

## Authors’ contributions

All authors read and approved the final manuscript. All the authors contributed equally to the case conception, design, interpretation and manuscript completion.
